# Population- and Individual-Level Dynamics of the Intestinal Microbiota of a Small Primate

**DOI:** 10.1128/AEM.00559-16

**Published:** 2016-05-31

**Authors:** Tuomas Aivelo, Juha Laakkonen, Jukka Jernvall

**Affiliations:** a Institute of Biotechnology, University of Helsinki, Helsinki, Finland; b Department of Veterinary Biosciences, University of Helsinki, Helsinki, Finland; University of Michigan

## Abstract

Longitudinal sampling for intestinal microbiota in wild animals is difficult, leading to a lack of information on bacterial dynamics occurring in nature. We studied how the composition of microbiota communities changed temporally in free-ranging small primates, rufous mouse lemurs (Microcebus rufus). We marked and recaptured mouse lemurs during their mating season in Ranomafana National Park in southeastern mountainous rainforests of Madagascar for 2 years and determined the fecal microbiota compositions of these mouse lemurs with MiSeq sequencing. We collected 160 fecal samples from 71 animals and had two or more samples from 39 individuals. We found small, but statistically significant, effects of site and age on microbiota richness and diversity and effects of sex, year, and site on microbiota composition, while the within-year temporal trends were less clear. Within-host microbiota showed pervasive variation in intestinal bacterial community composition, especially during the second study year. We hypothesize that the biological properties of mouse lemurs, including their small body size and fast metabolism, may contribute to the temporal intraindividual-level variation, something that should be testable with more-extensive sampling regimes.

**IMPORTANCE** While microbiome research has blossomed in recent years, there is a lack of longitudinal studies on microbiome dynamics on free-ranging hosts. To fill this gap, we followed mouse lemurs, which are small heterothermic primates, for 2 years. Most studied animals have shown microbiota to be stable over the life span of host individuals, but some previous research also found ample within-host variation in microbiota composition. Our study used a larger sample size than previous studies and a study setting well suited to track within-host variation in free-ranging mammals. Despite the overall microbiota stability at the population level, the microbiota of individual mouse lemurs can show large-scale changes in composition in time periods as short as 2 days, suggesting caution in inferring individual-level patterns from population-level data.

## INTRODUCTION

The intestinal microbiota has been shown to be remarkably stable over the lifetime of primates (1). Microbiota acquired early in life can be inherited from the mother (2) or from other social contacts (1), although the composition of the microbiota is also affected by the genetics of the host (3). Later in life, the microbiota can change due to external factors, such as food (4–6) or pathogens (7, 8). Seasonal or nonseasonal environmental change can lead to drastic changes in an animal's microbiota through changes in the aforementioned external factors (9); however, in some cases, the microbiota has been shown to be resilient against habitat perturbations, infections, or changes in diet (10, 11). Yet, there is a general expectation, at least in humans, that intestinal microbiota is acquired after birth, and this microbiota stays similar in composition barring events, like pregnancy, illnesses, or major dietary shifts (12, 13).

Whereas mice and rats are common experimental animals for intestinal microbiota studies in the laboratory, the microbiota of free-living small mammals is much less well studied. Fecal collection is usually performed as terminal sampling in small mammals, making longitudinal sampling of the same individuals impossible. In contrast, wild primates are common study subjects in behavioral studies and increasingly also in microbiota research. Primate gut microbiota varies with the social environment (14), pathogens (7, 15, 16), dietary variation (17–19), and biogeography (19, 20). Nevertheless, we expect primate gut microbiotas to evolve and adapt to the different life histories of their hosts (21–23). Thus, both variation in microbiota and the response to this variation can differ from species to species. Although the number of longitudinal studies using nonhuman primates remains small, studies are starting to reveal also cases of high individual variation in microbiota composition (24, 25).

The aim of our longitudinal study was to study temporal variation in the intestinal microbiota of small mammals by marking-and-recapturing rufous mouse lemurs. Rufous mouse lemurs are small (mean weight, approximately 45 g) nocturnal primates that exhibit extensive heterothermy (26, 27), like many species in the Cheirogaleidae family. They can hibernate for extended periods and also experience torpor in short bouts, most likely before and after sunset during the coldest periods of the day (28, 29). Mouse lemurs are a suitable longitudinal study species, as the individual life span can be up to 9 years in the wild, and they present at least partial territoriality. Our research questions were (i) how large is intraindividual variation in microbiota composition compared to interindividual variation, (ii) are there patterns of variation in the microbiota composition over the trapping season and between years, and (iii) how do individual differences and environmental variables affect microbiota composition? Our null expectation was that the mouse lemur microbiota at the population level reflects dynamics at the individual level.

## MATERIALS AND METHODS

We collected fecal samples from September to November in 2012 and 2013 in Ranomafana National Park (RNP) in southeastern Madagascar (21°16′S latitude and 47°20′E longitude). RNP consists of primary and secondary forests situated on lowland to montane areas (500 to 1,500 m above sea level), and it is surrounded by a peripheral zone with restrictions on land use (30). Mouse lemurs have their mating period in October, and practically all females are gestating from October to January (31).

We collected samples from two different transects: inside the National Park in Talatakely and in the peripheral zone in the campsite of Centre Valbio. The Talatakely transect was in the secondary forest with continuous canopy, and it was heavily used by tourist groups, whereas the campsite transect was a heavily degraded area with both trees and bushes that were both endemic and nonendemic and without continuous canopy. Each night before sunset, we set 40 to 50 traps to catch mouse lemurs and collected the traps approximately 2 h after sunset. The trapping and feces collection procedures are detailed elsewhere (32). We microchipped all mouse lemurs to allow for identification and longitudinal surveying of individuals. We estimated the age with validated methods (33) and divided mouse lemurs into young (1 to 2 years), midage (3 to 4 years), and old (≥5 years) groups. The research was approved by the ethics committee of Viikki Campus, University of Helsinki, Helsinki, Finland, and by the trilateral commission (CAFF/CORE) in Madagascar (permit 203/12/MEF/SG/DGF/DCB.SAP/SCBSE).

We could not measure mouse lemur body temperature and thus cannot directly assess their level of heterothermy. Nevertheless, mouse lemurs are known to be heterothermic during periods of inactivity and low ambient temperatures (29). We calculated the mean temperature for all trapping days while mouse lemurs are most likely in torpor (from 02h00 to 14h00). In addition, we calculated the monthly mean temperatures and total rainfall based on weather data (http://www.teamnetwork.org/data/query, data set 20150601041318_1609) we acquired from the Tropical Ecology Assessment and Monitoring (TEAM) network, which has weather stations run by Centre Valbio in the area (Table 1).

**TABLE 1:** Monthly rainfall accumulation and average temperature in Ranomafana during 2012 and 2013

Month	Temp (°C)		Rainfall (mm)	
	2012	2013	2012	2013
January	20.1	21.0	607	648
February	21.5	20.7	884	973
March	20.3	19.7	442	383
April	19.4	18.0	383	193
May	17.2	16.6	200	187
June	15.6	13.1	217	194
July	14.8	13.2	62	112
August	15.7	12.9	77	265
September	16.5	16.1	221	5
October	18.7	16.7	117	375
November	18.9	19.8	376	213
December	20.0	19.4	259	362

We obtained approximately 0.1 to 0.2 g of feces per sample and stored the feces in 1.5 ml of RNA*later* (Ambion, Inc., Austin, TX, USA) in −18°C. DNA was isolated with the PowerSoil DNA isolation kit (Mo Bio Laboratories, Inc., Carlsbad, CA, USA), according to the standard protocol. We amplified the 16S gene in V1-V2 region with primer pairs pA_Illum_FP_1 (ATCTACACTCTTTCCCTACACGACGCTCTTCCGATCTAGAGTTTGATCMTGGCTCAG) and pA_Illum_RP_1 (GTGACTGGAGTTCAGACGTGTGCTCTTCCGATCTGTATTACCGCGGCTGCTG), pA_Illum_FP_2 ATCTACACTCTTTCCCTACACGACGCTCTTCCGATCTTAGAGAGTTTGATCMTGGCTCAG) and pA_Illum_RP_2 GTGACTGGAGTTCAGACGTGTGCTCTTCCGATCTCGTATTACCGCGGCTGCTG), and pA_Illum_FP_3 ATCTACACTCTTTCCCTACACGACGCTCTTCCGATCTCTCTAGAGTTTGATCMTGGCTCAG) and pA_Illum_RP_3 GTGACTGGAGTTCAGACGTGTGCTCTTCCGATCTTAGTATTACCGCGGCTGCTG) and Phusion enzyme (New England BioLabs, Inc., Ipswich, MA, USA). The PCR protocol included an initial denaturation at 98°C for 30 s and 15 cycles of denaturation at 98°C for 10 s, annealing at 62°C for 15 s, and extension at 72°C for 15 s, followed by 10 min of final extension at 72°C. We verified the success of amplification by gel electrophoresis. For each sample, two replicates were separately amplified and then pooled. The amplicons were paired-end sequenced with the Illumina MiSeq platform in the sequencing facility of the Institute of Biotechnology, University of Helsinki.

Samples were isolated and amplified in 7 different batches and sequenced in 2 different batches. The amplification was performed in numerical order of the samples, which corresponded to chronological order, and sequencing was performed partly in chronological order: late samples from 2012 and early samples from 2013 were sequenced together, and early samples from 2012 and late samples from 2013 were sequenced together.

The amplicon sequences were demultiplexed, and subsequent sequence processing was performed using the mothur pipeline, along standard operating procedures (SOP) (34; http://www.mothur.org/wiki/MiSeq_SOP) when not otherwise mentioned. To purge unsuccessful contigs, only contigs between 439 and 511 bp were retained. The alignment was made against aligned SILVA bacterial references (release 102). Preclustering of the sequences was performed with a maximum difference of 5 bp. To reduce the number of unique sequences, all unique sequences composed of only one sequence were discarded. Sequences were classified by using Bayesian classifier with a training set (version 9) from the Ribosomal Database Project (35; http://rdp.cme.msu.edu). We used 97% similarity to determine the operational taxonomic units (OTUs).

We performed the initial statistical analysis in line with MiSeq SOP with mothur. We rarified the amplicons to the lowest sample size, 4,316 sequences. We estimated the alpha diversity of the samples with the inverse Simpson diversity index. Both the richness, defined as the number of OTUs per sample, and the Simpson diversity were normally distributed and homoscedastic. To explore the structures of communities, we calculated the Yue & Clayton (36) dissimilarity metric based on the proportions of OTUs in different samples and the Jaccard distance from presence-absence data (37). We used Jaccard distance, as it is a widely used intuitive metric, and the Yue & Clayton dissimilarity metric, as it takes into account (in comparison to widely used Bray-Curtis dissimilarity) both shared and nonshared species in each population and emphasizes the shared species with similar species proportions in communities. We visualized the dissimilarity metrics with nonmetric multidimensional scaling (NMDS), using three dimensions. To calculate how many community types there are in the samples, we used the Dirichlet multinomial mixtures method in mothur with Laplace approximation (38). The method describes communities as a vector of taxon probabilities created from Dirichlet mixture components. Mixture components are clustered, and the fit is evaluated with Laplace approximation.

For subsequent analysis, we used R (39) with the vegan package (40). We created repeated-measures analysis of variance models for rank-transformed richness counts and diversity indices. For each model, we began with a full model including interactions and simplified models, where terms that were not significant were removed until we were left with variables with a *P* value of <0.05. For the remaining analysis, we considered the variables site, age, year, week, and sex. There was no significant effect with sequencing batches, but we found a significant effect on isolation and amplification batches, and these batches were also included as variables in subsequent analysis. As *post hoc* tests for multivariate repeated-measure analysis of variance (ANOVA) are complex, we investigated the differences graphically. Nevertheless, we also performed *t* test comparisons for significant variables for diversity and corrected them with the Holm-Bonferroni method.

We tested if the communities overlap using permutational multivariate ANOVA (MANOVA) with dissimilarity matrices (41), taking into account the repeated sampling. Permutational MANOVA measures whether the groups are significantly different from each other. We also performed an analysis of multivariate homogeneity of group dispersions (42), which tells us if the within-group variation differs between groups. As analysis of multivariate homogeneity cannot be performed with multiple variables, we divided our samples into eight groups based on site, sex, and year and performed pairwise *post hoc* comparisons. To take repeated measures into account, we randomly sampled one sample per individual for analysis with 100 iterations.

To study the temporal effects on microbiota, we calculated variation in population-level and individual-level dissimilarity and used the Mann-Whitney U test to test dissimilarities between trapping years. For the individuals from which we had more than two samples, we plotted the proportions of bacterial phyla and the most common 20 OTUs on graphs for visual inspection. Additionally, we plotted NMDS axis loadings as a function of time and calculated and plotted the dissimilarity indices as a function of the temporal distance of samplings to see if there are temporal trends in the microbiota within the host. The dissimilarity indices were normally distributed; therefore, we explored significant variables by performing repeated-measures ANOVA on dissimilarity indices for mouse lemurs we caught several times as the response variable; the number of days between trapping, temperature, trapping year, site, sex, and age as explanatory variables; and mouse lemur individuals as the repeated factor.

We used several methods to explore which OTUs are driving the trends between groups. First, we chose all the OTUs that are present in >10% of the samples and, as they are not normally distributed, we used rank-transformed repeated-measures ANOVA to see if they differed significantly between the groups. Second, we used Dufrêne-Legendre indicator analysis, which uses the maximum indicator value among the groups as a test statistic, without the need for multiple tests (43). To further explore the difference between years, we performed a random forest approach with R package randomForest (44) to assess how well trapping year can be predicted from the community composition. We also used the mean decrease in accuracy as a proxy for the importance of single OTUs in differentiating separate groups.

### Accession numbers.

Raw data on the sampled individuals have been submitted to FigShare (http://dx.doi.org/10.6084/m9.figshare.1558204), and all sequence data have been deposited to the Sequence Read Archive under accession no. SRP063971.

## RESULTS

We collected a total of 176 samples for which the sequencing was successful (>1,000 amplicons; 8 unsuccessful sequencings had an amplicon number from 35 to 741), and we had complete metadata for 161 samples from 70 animals. Eighty-eight samples from 46 individuals were from 2012, and 73 samples from 36 individuals were from 2013, with 12 individuals caught in both years.

The total number of sequences after assembling the contigs was 14,283,388, and after the quality control, we had 7,417,887 sequences in total, presenting 77,032 unique sequences. Most of the discarded sequences had ambiguous bases or they were not assembled well (4,895,601 sequences), but they also included sequences that did not align properly (1,745,859), nonbacterial OTUs (96,427), and chimeras (127,614). There was a median of 36,915 quality-controlled amplicons per sample, with a standard deviation of 22,747. These sequences generated 4,524 bacterial OTUs. The median number of OTUs per sample was 257 (95% confidence interval [CI], 133 to 329 OTUs). Good's coverage estimator was ≥0.97 for each rarified sample.

### Stability of mouse lemur microbiota within trapping season.

**FIG 1:**
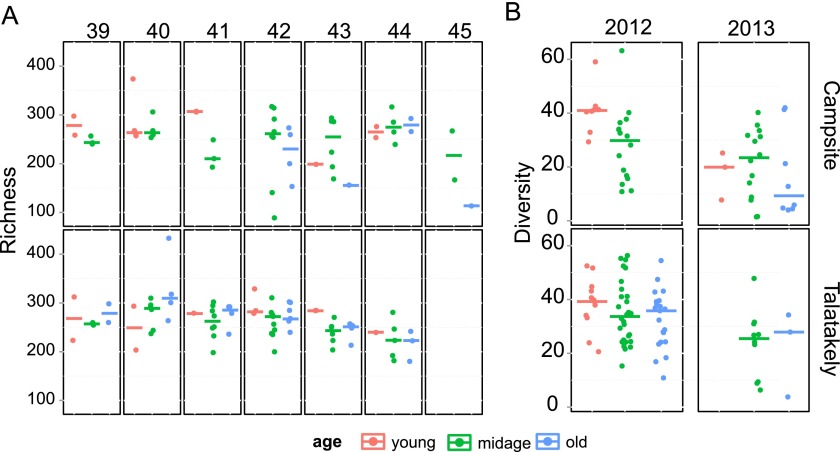
Richness and diversity in different age groups on two sites. (A) Richness is shown along the trapping weeks. Whereas the secondary-forest transect in Talatakely had rather stable richness, with a slight trend toward decreasing, the degraded transect in campsite had higher variation, which partly can be explained by the smaller sample size. The richness was statistically significantly lower in campsite than that in Talatakely. Different age groups have differing temporal trends: for example, old individuals have their peak richness earlier in the season than young individuals. (B) Inverse Simpson indices for different age groups in two sites in 2012 and 2013. The higher inverse Simpson index values indicate higher diversity. The graphs show a similar trend as in panel A, in that the diversity is more stable between age groups and years in a better-quality habitat (Talatakely) than in a degraded habitat (campsite).

For richness, the simplified model included site and interaction between age and week as significant variables. The ages had differing trends along the trapping season in Talatakely: old and midage mouse lemurs peaked in their microbiota richness in early season, whereas young individuals did not have a clear trend. There were fewer old individuals trapped in the campsite with no detectable trend. Overall, Talatakely had higher richness than the degraded campsite transect (Fig. 1A; see also Table S1 in the supplemental material). In comparison, the Simpson diversity indices, reflecting the diversity of bacterial OTUs in the intestinal microbiota, were explained statistically significantly by age, year, and site variables (see Tables S2 and S3 in the supplemental material), and there were no within-trapping season differences.

**FIG 2:**
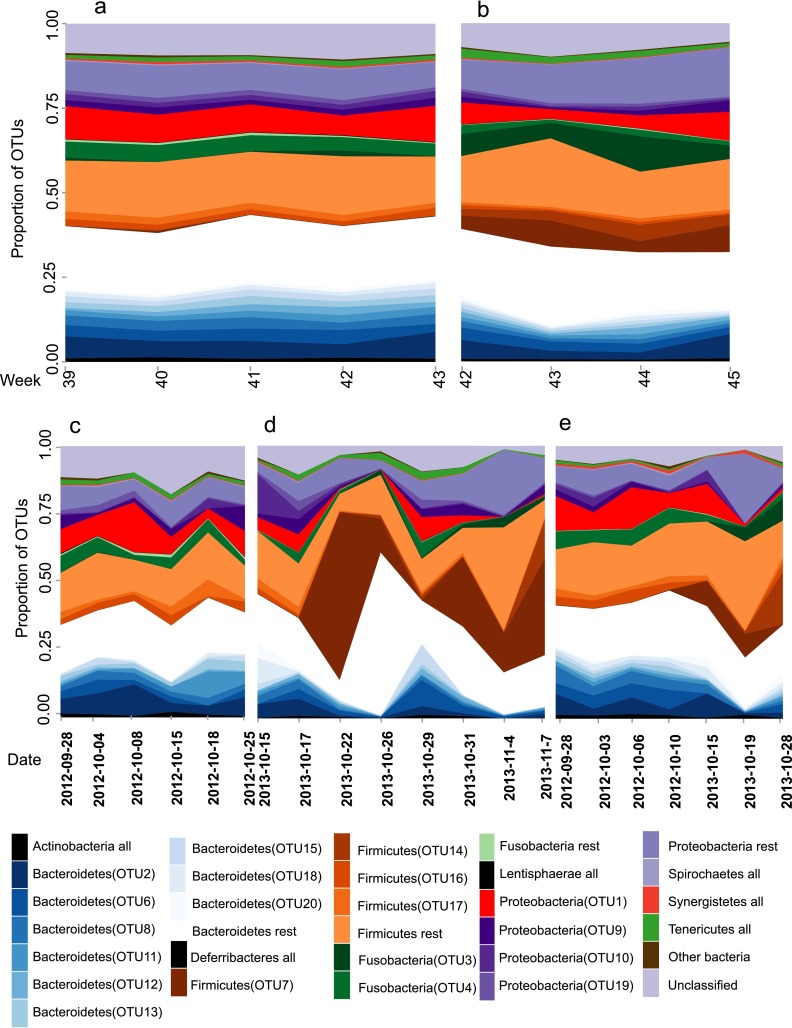
The bacterial composition in the whole mouse lemur population along the trapping season in years 2012 (a) and 2013 (b). The microbiota composition is shown weekly as average values across individuals. The microbiota is slightly different between years (e.g., OTU3, 7, and 14), but the overall patterns are remarkably stable over time. In comparison, microbiota in individual mouse lemurs varied substantially in 2013 but much less in 2012. All samples from the mouse lemur named Napolean (c) are from 2012, and the proportion of OTUs varies much less than that in Alan (d), from which all samples came in 2013. For Rachootin (e), we have samples for both years, and the microbiota composition follows the yearly trends. The graphs for the rest of the lemurs caught at least three times can be found in the Fig. S4 in the supplemental material. Dates in panels c to e are displayed in the format year-month-day.

During both trapping periods, 2012 and 2013, the composition of microbiota on the level of host population seemed to be stable (Fig. 2a and b), with no clear trends. In 2012, the weekly OTU profiles were very similar (Yue-Clayton [YC], 0.25 ± 0.05), whereas in 2013, the dissimilarities were larger (YC, 0.52 ± 0.09; Mann-Whitney U, U = 124, *P* < 0.001). In contrast, the temporal variation in the microbiota of individual mouse lemurs was more pronounced, even when the samples were taken on subsequent days (Fig. 2c to e): the median dissimilarities between samples of the same individuals were 0.40 ± 0.09 and 0.61 ± 0.12 in 2012 and 2013, respectively. Thus, the intestinal microbiota compositions were more stable in 2012 than in 2013 (Mann-Whitney U, U = 613, *P* < 0.001). The Jaccard index produced similar results (see Table S4 in the supplemental material).

We did not find any within-year temporal trend (see Fig. S1 in the supplemental material) with nonmetric multidimensional scaling of the bacterial communities. The stress values of NMDS were approximately 0.23 and 0.24, with *R*^2^ values 0.68 and 0.53 for Clayton-Yue and Jaccard distances, respectively. The stress values indicate mediocre goodness-of-fit with three dimensions (45), whereas the *R*^2^ values represent satisfying proportions of explained variation.

### Differences in microbiome between trapping seasons.

Although there were no differences in microbiota richness between years, the Simpson diversity was significantly lower in 2013 than it was in 2012 (Fig. 1B). In 2013, Talatakely had higher diversity than that of the campsite, whereas in 2012, diversities were similar in the two sites (see Table S3 in the supplemental material).

**FIG 3:**
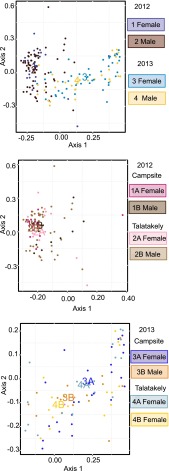
Visualization of the analysis of multivariate homogeneity of group dispersions based on Yue-Clayton dissimilarity metrics. There is a clear difference between years, whereas within-year groups overlap extensively. The between-group variation is smaller in 2012, as the group centroids are more spread apart in 2013.

For permutational multivariate analysis of dissimilarity matrices, the only significant variable was year, and the proportion of the explained variation was low (for Yue-Clayton, *P* = 0.010, *R*^2^ = 0.089; see Table S5 in the supplemental material; for Jaccard, *P* = 0.010, *R*^2^ = 0.064; see Table S6 in the supplemental material). Analysis of multivariate homogeneity of group dispersions showed most clearly the distinction between years, whereas the effects of sex and site were not as clear (Fig. 3; see also Fig. S2 and Tables S7 and S8 in the supplemental material). The main effect of the year was corroborated by Laplace approximation of having two distinct partitions: all individuals in 2012 and 49.4% of the individuals from year 2013 belong to the first partition, whereas the rest of the individuals from year 2013 belong to the other partition. Of the lemurs that had been trapped both years, 6 out of 12 changed their partition.

The presence-absence data for three OTUs were significantly different between the two trapping years: these were unclassified members of the families Pasteurellaceae (prevalences in 2012 and 2013 were 3.3% and 83.5%, respectively, and the proportions of all OTUs in 2012 and 2013 were <0.1% and 1.8%, respectively) and Prevotellaceae (prevalences, 1.0% versus 84.6%; proportions, <0.1% versus 1.2%, respectively) and the phylum Proteobacteria (prevalences, 58.2% versus 2.6% for 2012 and 2013, respectively; proportions, 0.2% versus <0.1%, respectively). Dufrêne-Legendre indicator analysis resulted in 266 differentially associated OTUs, which also included three previously mentioned OTUs. Of the 20 most common OTUs, these included Fusobacteriaceae (OTU3 in Fig. 2: prevalence, 27.1% versus 86.7% for 2012 and 2013, respectively; proportion, 0.5% versus 4.9%, respectively), Lactococcus (OTU7: prevalence, 50.5% versus 86.7%, respectively; proportion, 0.1% versus 3.7%, respectively), and Clostridium cluster XIva (OTU14: prevalence, 100% versus 76.5%, respectively; proportion, 1.5% versus 2.4%, respectively). A random forest algorithm returned an out-of-bag error rate of 9.4%, which means the year of sampling cannot be reliably predicted from OTU composition. The mean decrease in accuracy was highest in the previously mentioned OTUs labeled as Prevotellaceae (40.4%) and Pasteurellaceae (36.4%).

Only 8 OTUs were present in every sample; however, 83 OTUs were present in at least 90% of the samples. Nevertheless, these 83 OTUs represent only 50.7% of all sequences. Thus, the core bacteria represent only half of all available bacteria. This picture looks different, though, if we divide the analysis by years. In 2012, 35 OTUs were present in all samples and 101 OTUs in at least 90% of the samples. These OTUs represented 43.0% and 65.3% of all sequences, respectively. In 2013, there were only 15 OTUs in all samples and 47 OTUs in at least 90% of the samples, and these OTUs represented 6.7% and 33.9% of all sequences, respectively.

### Individual differences in microbiota variation.

While there were no significant differences between individuals in richness, the within-subject test indicated that there is a significant effect of age on alpha diversity. The highest diversities were recorded in 2012 in young individuals at both sites. In 2013, the old individuals had low diversity at the campsite, whereas the diversity at Talatakely was comparable to that with midage individuals (Fig. 1; see also Table S3 in the supplemental material). The number of individuals with an estimated age was lower in 2013, and there was only one young individual available. There were six midage individuals and three old individuals at each site.

**FIG 4:**
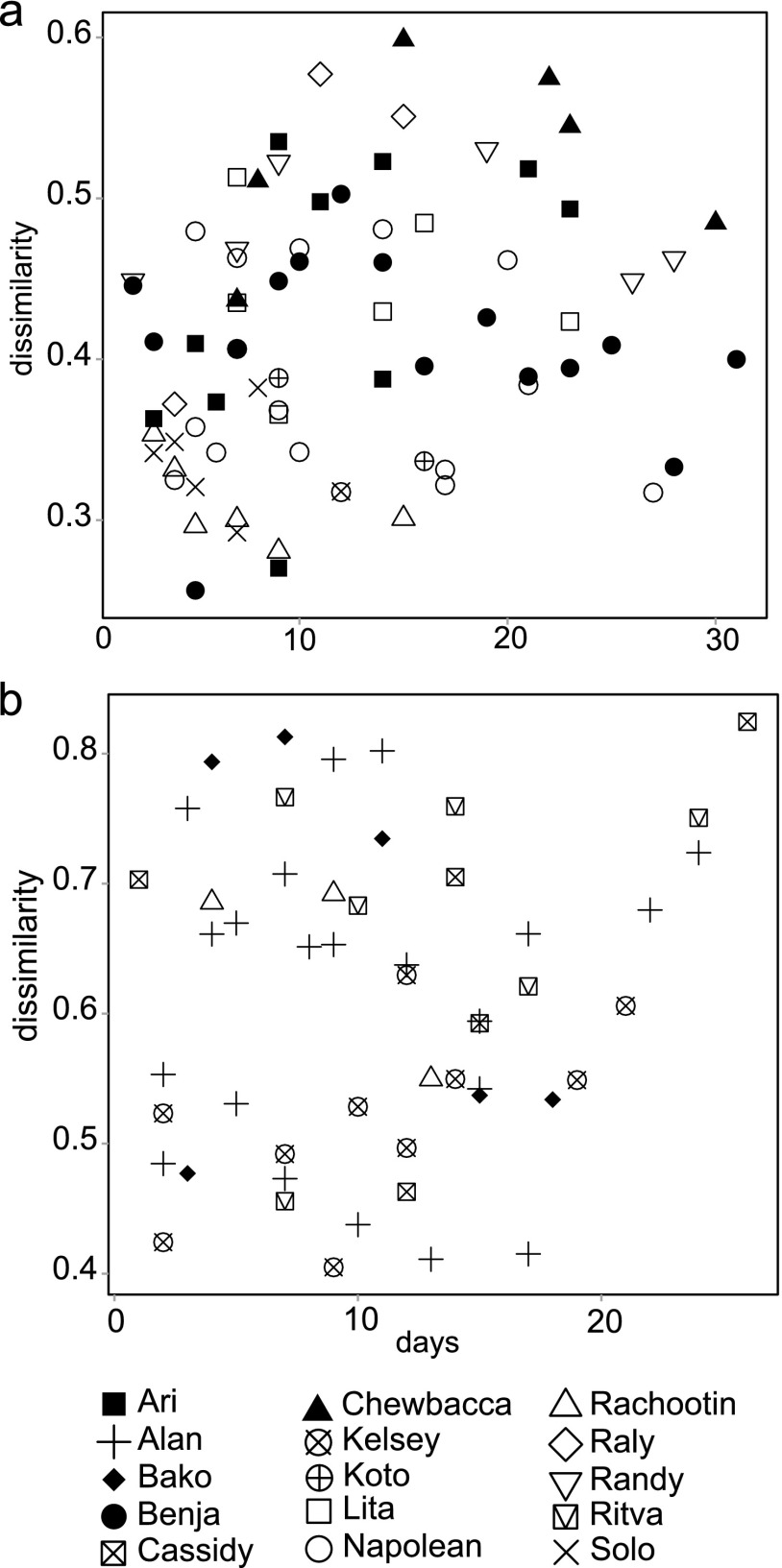
Dissimilarity indices of microbiota from mouse lemurs caught three or more times as a function of the intervals of the trappings in 2012 (a) and 2013 (b), with Yue-Clayton dissimilarity index. There is no clear trend, but the dissimilarities between samples from the same individual are bigger in 2013 than those in 2012.

We plotted the dissimilarities of the microbiome communities in individuals we caught several times (Fig. 4; see also Fig. S1 in the supplemental material). This shows that even with the shortest trapping intervals (1 day), the dissimilarity can be quite high (0.7 in Alan in 2013). Some mouse lemurs have consistently lower variation in their microbiome (e.g., Rachootin in 2012 and Kelsey in 2013), while others have high dissimilarity between samples (e.g., Chewbacca in 2012). There are also mouse lemurs with variation in the level of variation in microbiome composition (e.g., Alan in 2013). The dissimilarity indices were similar with Jaccard index (see Fig. S3 in the supplemental material).

In repeated-measures ANOVA on dissimilarities in mouse lemurs we sampled more than once, there were five statistically significant variables: sex, site, year, mean temperature, and days between samplings, although the coefficients were comparatively low for variables other than year (Table 2). Year 2013 had substantially higher dissimilarities than those in 2012, indicating more changes in microbiota composition. Males and individuals from Talatakely had lower dissimilarities, whereas the lower mean temperature and longer temporal distance between trappings also resulted in higher dissimilarity between microbiota communities. The results were similar to the Jaccard index, with the exception that the interval of samplings in days was not significant (see Table S8 in the supplemental material).

**TABLE 2:** Analysis of variance in Yue-Clayton dissimilarity index with statistically significant variables

Parameter	df	Sum of squares	Mean sum of squares	*F*	*P*	Specific variable	
						Parameter	Coefficient
Temp	1	0.2237	0.2237	12.969	0.001	Temperature	−0.012
Sex	1	0.4556	0.4556	26.406	<0.001	Male	−0.053
Site	1	0.2024	0.2024	11.733	0.002	Talatakely	−0.014
Yr	1	0.3604	0.3604	20.890	<0.001	2013	0.164
Days	1	0.1470	0.1470	8.523	0.007	Days	0.011
Residuals	27	0.4658	0.0173			Intercept	0.497

## DISCUSSION

At the individual level, our results indicate that the intestinal microbiota of individual mouse lemurs is dynamic. In the first year, individuals exhibited limited temporal variation in microbiota composition; however, changes were more pervasive in the second year (Fig. 2d and e). Consequently, alpha diversity, but not richness, in microbiota was lower in the second year (Fig. 1). The difference in beta diversity between years was not only due to changes in species proportions but also due to the absence of shared bacterial OTUs.

In other primates, the microbiota has been considered to be more stable than what we found in mouse lemurs (19, 25), although wild baboons also seem to have highly varied microbiota (24). Nevertheless, there have not been previous studies with wild cheirogaleids.

In contrast to the dynamic variation at the individual level, variation at the population level was relatively subtle; in fact, in year 2012, the population-level composition of gut microbiota was very stable (Fig. 2a and b). This dynamic is very similar to that found in our previous study with mouse lemur parasites, in which the abundant putative parasite species were rather stable in the population-level data, yet the turnover in individual hosts was fast (32). Our results are comparable to widespread intraindividual variation in deer mice (Peromyscus spp.) (46) but contrast with that with wild wood mice (Apodemus sylvaticus), which had consistent seasonal variation (9). Although the intra- and interindividual variations were pervasive, we found a distinct core set of microbial OTUs present in all individuals within 1 year, as was also found in a study on Peromyscus (46).

There are several potential reasons for the high intraindividual variation of the microbiota of mouse lemurs. First, the small size of these mammals might lead to faster changes in the microbiota, as it leads to a potentially faster metabolism and shorter transit time in the intestine. Second, solitary hosts, like mouse lemurs, are expected to have higher intraindividual variation in the activity of immune defense, and as the microbiota can be modulated by immune defense (47), this might cause temporal variation in microbiota. Third, as nutrition is an important source of pathogens, omnivores and generalists, like mouse lemurs, should have immune responses different from those of specialist foragers, as they are expected to encounter more pathogens (47). Fourth, Madagascar is known for its harsh and unpredictable climate (48), and microbiota variation can also be an adaptation to these environments with, e.g., changes in diet. Fifth, mouse lemurs are heterothermic animals (49), and torpor can affect the microbiota. Indeed, we found that lower mean temperature during the most probable torpor time increased variation in gut microbiota composition. Furthermore, in 2013, the average temperature was continuously lower than that during 2012 (Table 1), indicating that the high variation of microbiota composition in 2013 (Table 2 and Fig. 2) might be partly explained by lower temperatures inducing higher body temperature variation in mouse lemurs. This possibility remains speculative, however, as we do not know whether lower ambient temperature is reflected in mouse lemur body temperature. Previous studies with heterothermic mammals have shown that ambient temperature does not affect the microbial composition, but the key determinant to composition is the diet (arctic ground squirrels [50] and 13-lined ground squirrels [51]). Nevertheless, fasting and hibernation seem to have separate effects on microbiota (52). In previous studies, the hibernators had a lower abundance of Firmicutes and lactobacilli and higher abundance of Bacteroidetes and Proteobacteria, whereas in our data, in 2013, the two clearly most abundant OTUs were Fusobacteriaceae (OTU3 in Fig. 2) and Lactococcus (OTU7). It should be noted that previous studies used terminal sampling and thus prevented longitudinal sampling of the same individuals.

The variation in bacterial community composition varied itself between host individuals, as some individuals had consistently lower variation than others (Fig. 4). Higher age correlates with higher diversity and richness of microbiota but not with variation. Previous studies with primates have shown differing patterns: older baboons had higher diversity than juveniles (24), whereas in chimpanzees, the juveniles had higher diversity (1), and diversity in black howler monkeys was not differentiated according to age (21). Our samples did not show differences in microbiota composition among the different-aged individuals, but it should be noted we did not sample juvenile mouse lemurs, and thus we would not expect drastic differences between age classes (13). Female lemurs and lemurs at the campsite had a slightly more varied microbiota (see Tables S7 and S8 in the supplemental material), although these effects are small compared to the intraindividual variation and the difference between years. In comparison to chimpanzees and mouse lemurs, sex does not have an effect on microbiota composition in baboons and black howler monkeys (14, 17, 24, and this study). Higher variability in females could be explained by our trapping coinciding with the start of the gestation period, which has been shown to alter microbiota (1). The Talatakely transect is composed of secondary forest, whereas the campsite transect is a highly degraded area. In our previous work, we showed that the body condition of mouse lemurs is lower at the campsite than at Talatakely (53). Thus, the habitat quality may lead to lower variation in microbiota composition (see Table S8), and it also correlates positively with microbiota diversity and species richness (Fig. 1 and Table 2). It has been shown that monkeys in degraded habitats have a less diverse diet, and thus their microbiomes were also less diverse (17, 54), but variations in diversity can also be driven by more contact with humans, livestock, and other companion animals and stress induced by disturbance (55).

Our results with respect to the high variability at the individual level may in part be due to the rather low number of mouse lemur individuals caught several times. Nevertheless, it is noteworthy that the population-level analysis provided more stable patterns than the longitudinal sampling of individuals. This also means that the difference between years can be driven in part by interindividual differences, as we sampled partially different sets of lemurs (i.e., some lemurs were included in only one of the two years and others were included in both years). We did not have age data for all individuals in 2013, and thus there can also be differences in the ratios of mouse lemur age classes in 2 years for which we cannot account. Although richness and diversity were not statistically significantly different between years in within-subject tests of repeated-measures ANOVA (see Tables S1 to S3 in the supplemental material), the microbiota composition did differ (see Tables S5 and S6 in the supplemental material), and thus time also drives intraindividual variation. We also trapped mouse lemurs for only 2 years and thus cannot draw definitive conclusions on the stability of mouse lemur gut microbiota.

In conclusion, the microbiota of mouse lemurs seem to show greater variation at the individual lemur level than at the population level. The difference in the overall amount of variation between two trapping years suggests there are extrinsic factors that cause the variation. As the year with higher intraindividual variation had a lower mean temperature during the trapping season, we propose that small body size together with the heterothermy of mouse lemurs could be a contributing factor for the variation in microbiota stability. Nevertheless, more longitudinal studies are needed to find factors affecting the year-to-year variation in mammal microbiota.

## Supplementary Material

Supplemental material
