# Supplementary material for: Population- and Individual-Level Dynamics of the Intestinal Microbiota of a Small Primate
Source: Appl Environ Microbiol. 2016 May 31;82(12):3537–45. doi: 10.1128/AEM.00559-16 (PMC4959157; doi:10.1128/AEM.00559-16)
Supplement: Supplemental material [file supp_82_12_3537__index.html]

Supplemental material 

# Population- and Individual-Level Dynamics of the Intestinal Microbiota of a Small Primate

## Supplemental material

- Supplemental file 1 -

  Model information, statistically significant variables for richness (Table S1) and diversity (Table S2) in the microbiome, pairwise comparisons for diversity (Table S3), stability of microbiota (Table S4), permutational MANOVA (Tables S5 and S6), analysis of multivariate homogeneity of group dispersion (Table S7), numbers of significant *P* values in *post hoc* tests (Table S8), analysis of variance in Jaccard dissimilarity index (Table S9), nonmetrical dimensional scaling loadings for dissimilarity metrics (Fig. S1), visualization of the analysis of multivariate homogeneity of group dispersions based on Jaccard dissimilarity metrics (Fig. S2), dissimilarity indices of microbiomes from mouse lemurs caught three or more times as a function of the intervals of the trappings (Fig. S3), and microbiome OTU compositions in the mouse lemurs caught at least three times (Fig. S4).

  PDF, 1.6M
