# Supplementary material for: Population- and Individual-Level Dynamics of the Intestinal Microbiota of a Small Primate
Source: Appl Environ Microbiol. 2016 May 31;82(12):3537–45. doi: 10.1128/AEM.00559-16 (PMC4959157; doi:10.1128/AEM.00559-16)
Supplement: Supplemental material [file AEM.00559-16_zam999117188so1.pdf]

Supplementary information

We estimated the alpha diversity of the samples with inverse Simpson diversity index and richness was used as rank-transformed number of OTUs. We considered following variables: PCR batch, sequencing batch, site, age, year, week and sex. We created repeated measures analysis of variance models for richness counts and diversity indices. For each model, we began with full model including interactions and simplified models by removing terms which we were not significant until we were left with variables with p-value < 0.05.

Post hoc multiple comparisons between different groups for diversity indices were done with multiple t-tests with Holm-Bonferroni correction (Table S3). We were not able to do similar analysis with richness as number of different weeks lead to underpowered comparisons, and thus these differences can only be explored via graphical means.

To explore the structures of communities, we calculated Yue & Clayton dissimilarity metric based on proportions of OTUs in different samples and Jaccard distance based on presence-absence data. We tested if the communities overlap using permutational manova with dissimilarity matrices taking into account the repeated sampling. We also performed analysis of multivariate homogeneity of group dispersions. As analysis of multivariate homogeneity cannot be performed with multiple variables, we divided our samples to eight groups on site, sex and year and performed pairwise post hoc comparisons. To take repeated measures into account, we sampled randomly one sample per individual for analysis with 100 iterations.

We built models by building first inclusive model and then leaving out all non-significant variables:

<u>Initial model for microbiome richness:</u>

richness ~pcrbatch:year+age*sex*week+year*site, with name as repeated measure

<u>Final model for microbiome richness (Table S1):</u>

richness ~ age*week+site, with name as repeated measure

<u>Initial model for microbiome diversity:</u>

diversity ~pcrbatch:year+age*sex*week+year*site, with name as repeated measure

<u>Final model for microbiome diversity (Table S2):</u>

diversity~ age+year+site, with name as repeated measure

<u>Initial model for permutational multivariate analysis of dissimilarity matrices:</u>

dissimilarity matrix ~ pcrbatch:year+age*sex*week+year*site, with name as repeated measure

<u>Final model for permutational multivariate analysis of dissimilarity matrices (Table S4 and Table S5):</u>

dissimilary matrix ~ age with name as repeated measure

<u>Initial model for repeated measures ANOVA on dissimilarities within-host:</u>

dissimilarity ~ age+sex+site+year+days + Error (name)

<u>Final model for repeated measures ANOVA on dissimilarities within-host with Yue-Clayton metric (Table 2):</u>

dissimilarity ~ sex+site+year+days + Error (name)

Final model for repeated measures ANOVA on dissimilarities within-host with Jaccard metric (Table S7):

dissimilarity ~ sex+site+year+ Error (name)

Supplementary Table 1: The statistically significant variables for richness in microbiome were age and site of mouse lemur and the trapping week. Also, the interaction between age and trapping week was significant.

error: name	df	Sum Sq	Mean Sq	F-value	p-value
age	3	21959	7320	4.237	0.00861
week	1	18643	18643	10.791	0.00167
site	2	16537	8269	4.786	0.01161
age*week	3	19669	6556	3.795	0.01444
Residuals	66	108835	1728		
**error: within**					
age	3	13101	4367	2.187	0.0953
week	1	487	487	0.244	0.6225
age*week	3	10247	3416	1.711	0.1708
Residuals	86	171698	1996		

Supplementary Table 2: The statistically significant variables for diversity in microbiome were age and site of mouse lemur and the trapping year.

error: name	df	Sum Sq	Mean Sq	F-value	p-value
age	3	28606	9535	5.769	0.00145
year	1	40317	40317	24.394	5.64e-06
site	2	17983	8992	5.440	0.00650
Residuals	66	109081	1653		
**error: within**					
age	3	24182	8061	4.531	0.00528
year	1	2687	2687	1.510	0.22232
Residuals	89	158322	1779		

Supplementary Table 3: Pairwise comparisons for diversity with abovementioned significant variables. The p-values are corrected with Holm-Bonferroni method. Statistcally significant differences are marked with grey background color.

			2012					2013			
			Campsite		Talatakely			Campsite			Talatakely
			young	mid-age	young	mid-age	old	young	mid-age	old	mid-age
2012	Campsite	mid-age	0.16								
2012	Talatakely	young	1.00	0.29							
2012	Talatakely	mid-age	1.00	1.00	1.00						
2012	Talatakely	old	1.00	1.00	1.00	1.00					
2013	Campsite	young	0.001	0.24	0.003	0.01	0.03				
2013	Campsite	mid-age	<0.001	1.00	0.001	0.003	0.03	1.00			
2013	Campsite	old	<0.001	0.15	<0.001	< 0.001	0.003	1.00	1.00		
2013	Talatakely	mid-age	0.59	1.00	1.00	1.00	1.00	0.18	0.82	0.12	
2013	Talatakely	old	1.00	1.00	1.00	1.00	1.00	0.10	0.43	0.10	1.00

Supplementary Table 4: The stability of microbiota in community level weekly averages and in individual-level sampling. Dissimilarities between samples were calculated with Jaccard index and significant differences by Mann-Whitney U.

	2012	2013	U	p
Weekly	0.40±0.06	0.67±0.09	87	< 0.001
Individual	0.56±0.11	0.75±0.13	213	< 0.001

Supplementary Table 5: Permutational manova with Yue-Clayton dissimilarity matrices. The only statistically significant variable was year.

	df	Sum Sq	Mean Sq	F.model	R2	p-value
year	1	4.461	4.4606	15.348	0.08854	0.009901
residuals	158	45.919	0.2902		0.91146	

Supplementary Table 6: Permutational manova with Jaccard dissimilarity matrices. The only statistically significant variable was year.

	df	Sum Sq	Mean Sq	F.model	R2	p-value
year	1	2.279	2.279	10.872	0.0644	0.009901
residuals	159	33.123	0.2096		0.9356	

Supplementary Table 7: Analysis of multivariate homogeneity of group dispersion. The table shows the number of significant p-values in post hoc tests after an iteration of 100 times on analysis of multivariate homogeneity of group dispersions with Yue-Clayton metric. There are rarely statistically significant differences within years. Females in Campsite in 2013 differs most from the rest of the samples, while females in Talatakely in 2013 and 2012 seem to differ much less.

			2012				2013		
			Campsite		Talatakely		Campsite		Talatakely
			F	M	F	M	F	M	F
2012	Campsite	M	0						
	Talatakely	F	0	0					
		M	4	2	0				
2013	Campsite	F	97	100	83	100			
		M	61	45	27	56	15		
	Talatakely	F	54	36	21	49	15	0	
		M	73	66	45	86	0	0	1

Supplementary Table 8: Number of significant p-values in post hoc tests after an iteration of 100 times on analysis of multivariate homogeneity of group dispersions with Jaccard distance. There are rarely statistically significant differences within years. Females in Campsite in 2013 differs most from the rest of the samples, while females in Talatakely in 2013 and 2012 seem to differ much less.

			2012				2013		
			Campsite		Talatakely		Campsite		Talatakely
			F	M	F	M	F	M	F
2012	Campsite	M	0						
	Talatakely	F	0	0					
		M	0	0	2				
2013	Campsite	F	92	99	81	100			
		M	14	28	7	30	13		
	Talatakely	F	26	42	15	57	10	0	
		M	59	88	44	100	0	1	4

Supplementary Table 9: Analysis of variance in Jaccard dissimilarity index with statistically significant variables. Coefficients of model are shown in the column in the right.

	df	Sum of squares	Mean sum of squares	F	p		Coefficients
Temperature	1	0.2775	0.2775	15.847	< 0.001	Temperature	-0.004
Sex	1	0.3858	0.3858	22.032	< 0.001	Male	-0.030
Site	1	0.1696	0.1696	9.683	0.004	Talatakely	-0.012
Year	1	0.4007	0.4007	22.80	< 0.001	2013	0.159
Residuals	28	0.4904	0.0175			Intercept	0.611

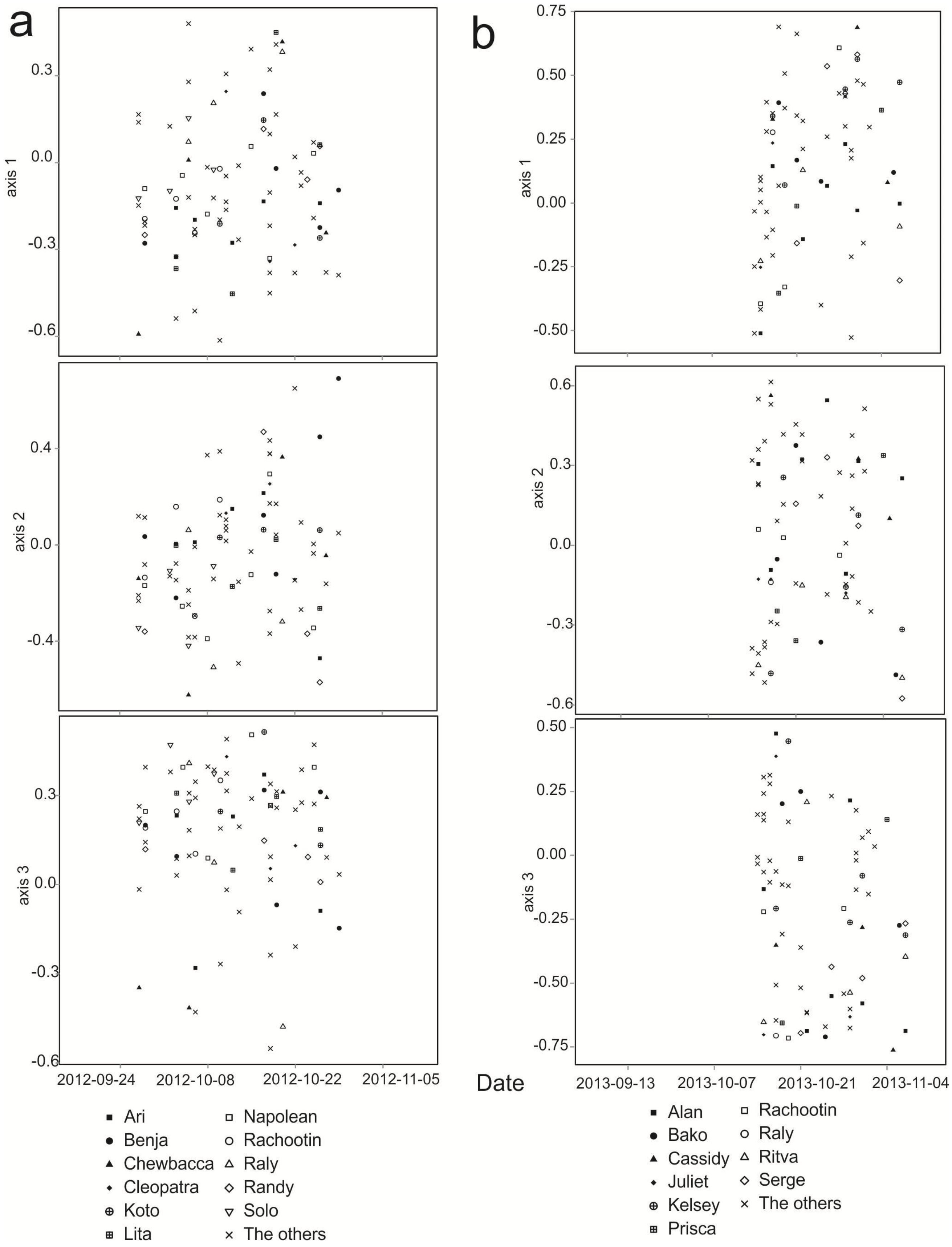
a
axis 1
axis 2
axis 3
0.3
0.0
-0.3
-0.6
0.4
0.0
-0.4
2012-09-24
2012-10-08
2012-10-22
2012-11-05
Ari
Benja
Chewbacca
Cleopatra
Koto
Lita
Napolean
Rachootin
Raly
Randy
Solo
The others
b
0.75
0.50
0.25
0.00
-0.25
-0.50
0.6
0.3
0.0
-0.3
-0.6
-0.75
Date
2013-09-13
2013-10-07
2013-10-21
2013-11-04
Alan
Bako
Cassidy
Juliet
Kelsey
Prisca
Rachootin
Raly
Ritva
Serge
The others

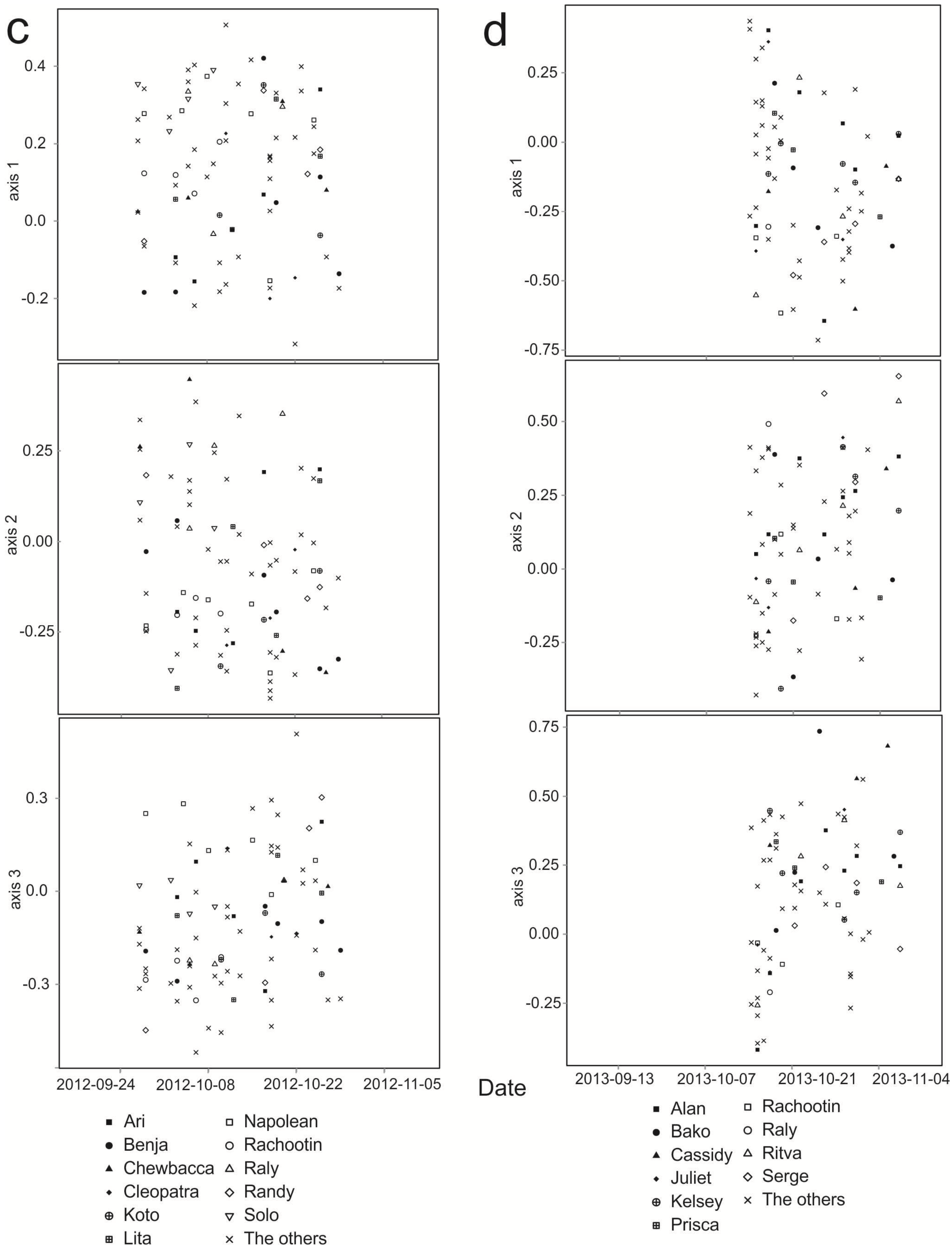

Supplementary Figure 1: Non-metrical dimensional scaling loadings for dissimilarity metrics for a) Yue-Clayton for 2012, b) Yue-Clayton for 2013, c) Jaccard index for 2012 and d) Jaccard index for 2013 as a function of time. The individuals with more than three samples per year have been named. There are three different dimensions from axis 1 to 3.

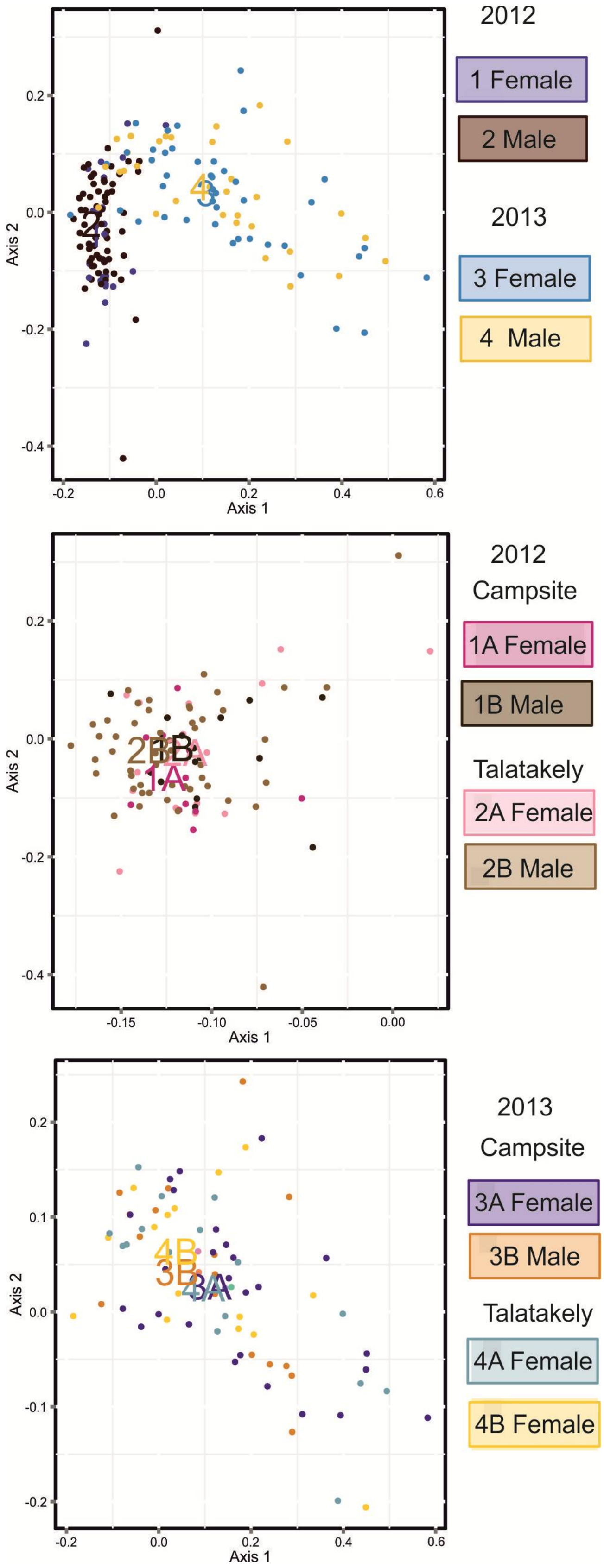

Supplementary Figure 2: Visualization of the analysis of multivariate homogeneity of group dispersions based on Jaccard dissimilarity metrics. There is a clear difference between years whereas within-year groups overlap extensively. The between-group variation is smaller in 2012 as the group centroids are more spread apart in 2013.

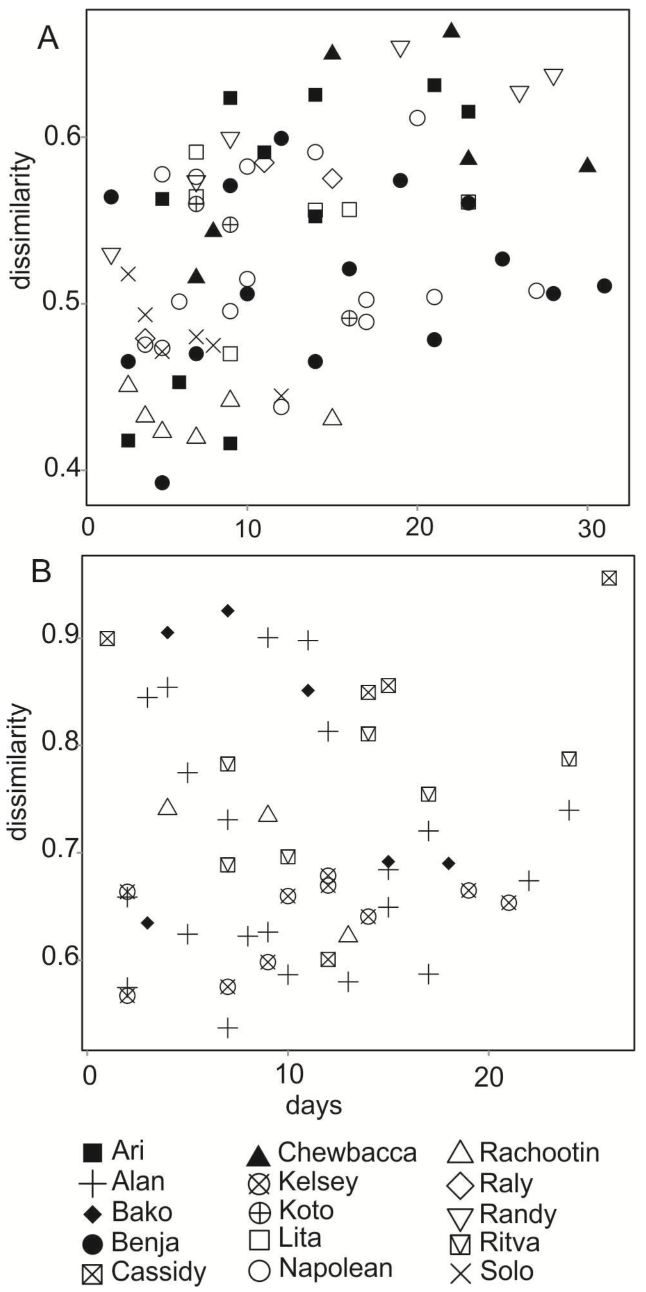

Supplementary Figure 3: Dissimilarity indices of microbiomes from mouse lemurs caught three or more times as a function of the intervals of the trappings in a) 2012 and b) 2013 with Jaccard dissimilarity index. The graphs are similar to Yue-Clayton dissimilarity indices.

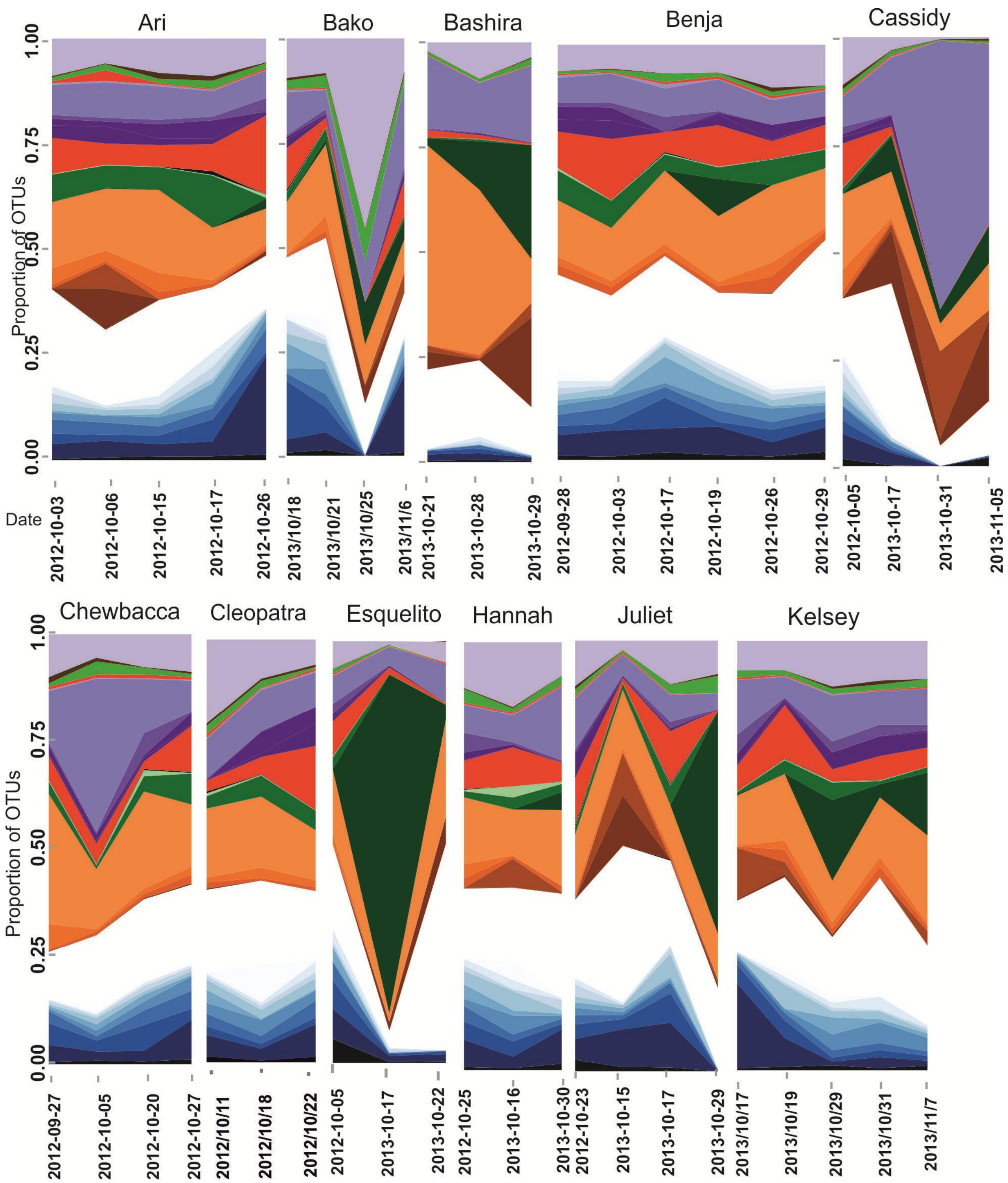
Ari
Bako
Bashira
Benja
Cassidy
Chewbacca
Cleopatra
Esquelito
Hannah
Juliet
Kelsey
Proportion of OTUs
0.00
0.25
0.50
0.75
1.00
Date
2012-10-03
2012-10-06
2012-10-15
2012-10-17
2012-10-26
2013/10/18
2013/10/21
2013/10/25
2013/11/6
2013-10-21
2013-10-28
2013-10-29
2012-09-28
2012-10-03
2012-10-17
2012-10-19
2012-10-26
2012-10-29
2012-10-05
2013-10-17
2013-10-31
2013-11-05
2012-09-27
2012-10-05
2012-10-20
2012-10-27
2012/10/11
2012/10/18
2012/10/22
2012-10-05
2013-10-17
2013-10-22
2012-10-25
2013-10-16
2013-10-30
2012-10-23
2013-10-15
2013-10-17
2013-10-29
2013/10/17
2013/10/19
2013/10/29
2013/10/31
2013/11/7

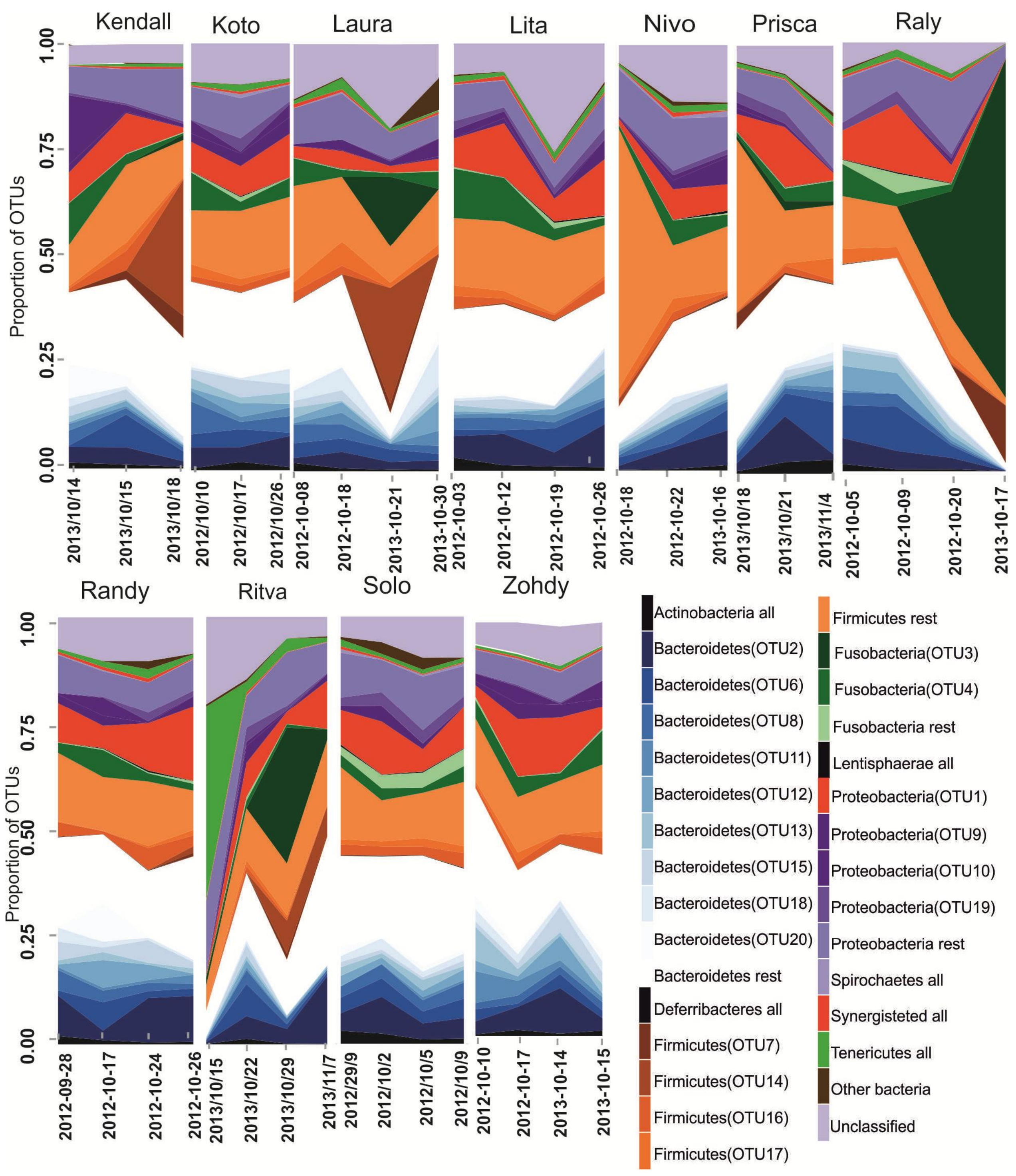

Supplemental Figure 4: The microbiome OTU compositions in the mouse lemurs caught at least three times.
